# Supplementary material for: Design, Synthesis, and Biological Evaluation of Pyridineamide Derivatives Containing a 1,2,3-Triazole Fragment as Type II c-Met Inhibitors
Source: Molecules. 2019 Dec 18;25(1):10. doi: 10.3390/molecules25010010 (PMC6983042; doi:10.3390/molecules25010010)
Supplement: Supplementary file 1 [file molecules-25-00010-s001.pdf]

Supplementary Materials:

Design, Synthesis and Biological Evaluation of Pyridineamide Derivatives Containing 1,2,3-triazole Fragment as Type II c-Met Inhibitors

Hehua Xiong [1,†], Jianxin Cheng [1,†], Jianqing Zhang [1], Qian Zhang [1], Zhen Xiao [1], Han Zhang [1], Qidong Tang [1,*] and Pengwu Zheng [1,*]

Content

^{1}H-NMR spectra of all target compounds **B1, B6, B12, B13, B20, B25** and **B26 (Figure 1-7).**

^{13}C-NMR spectra of compound **B6, B7, B10, B11, B14, B25** and **B26 (Figure 8-14).**

TOF MS spectra of compound **B13**, **B17**, **B19**, **B21**, **B22** and **B25 (Figure 15-20).**

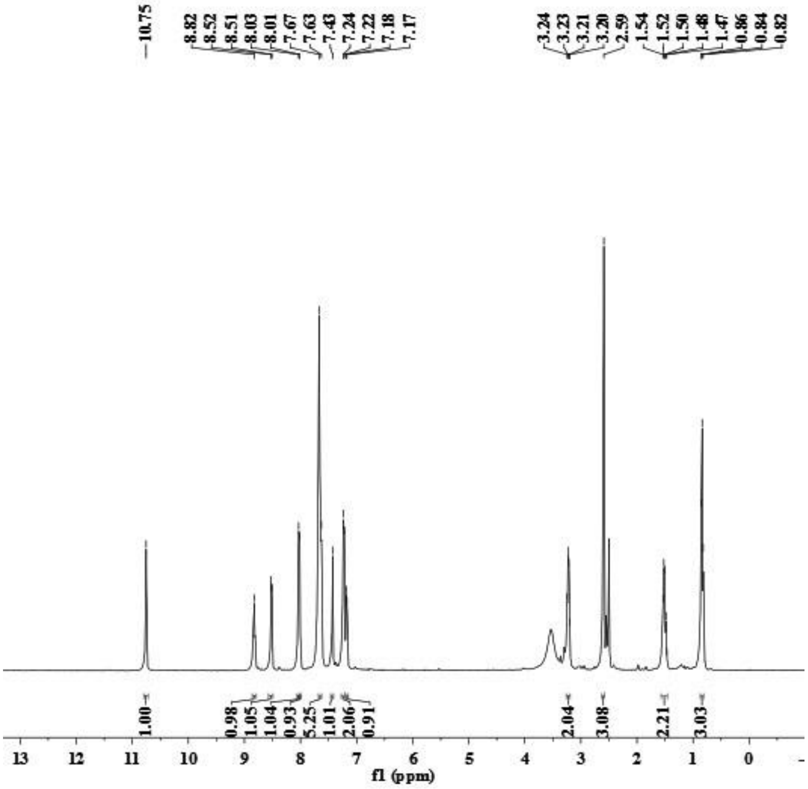

Figure 1. ^{1}H-NMR spectrum of compound **B1**

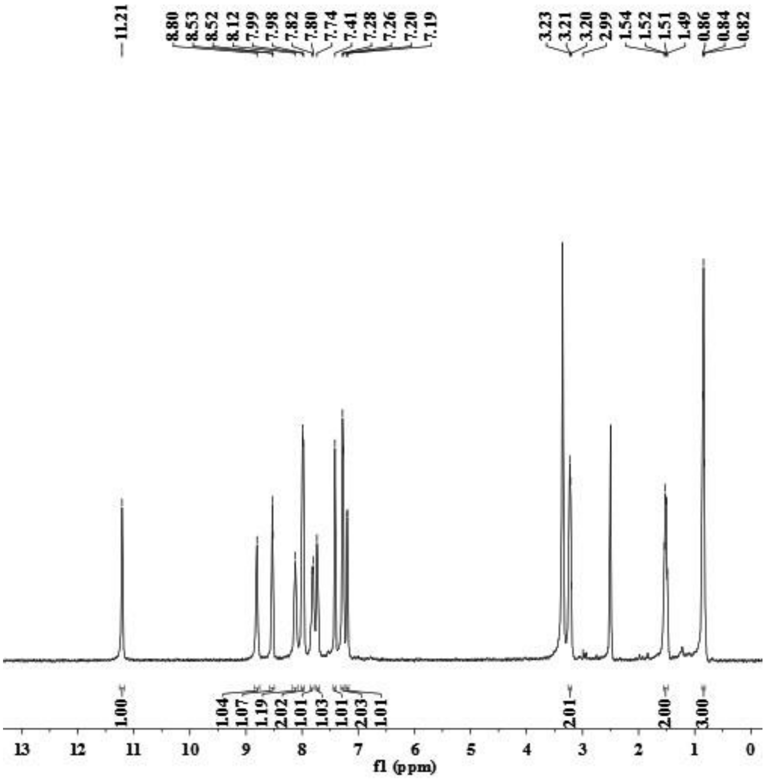

Figure 2. ^{1}H-NMR spectrum of compound **B6**

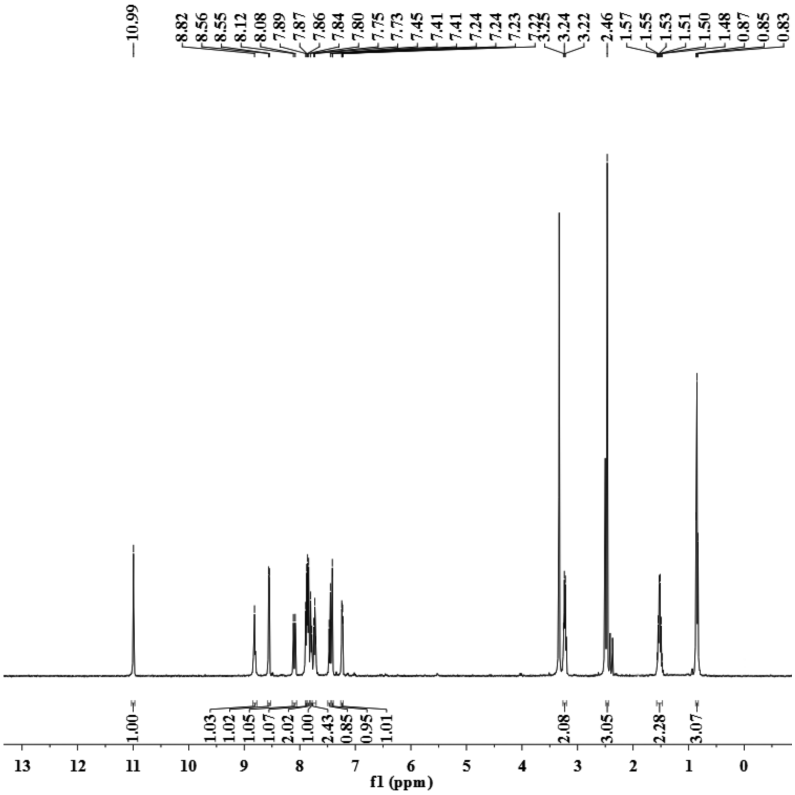

Figure 3. ^{1}H-NMR spectrum of compound **B12**

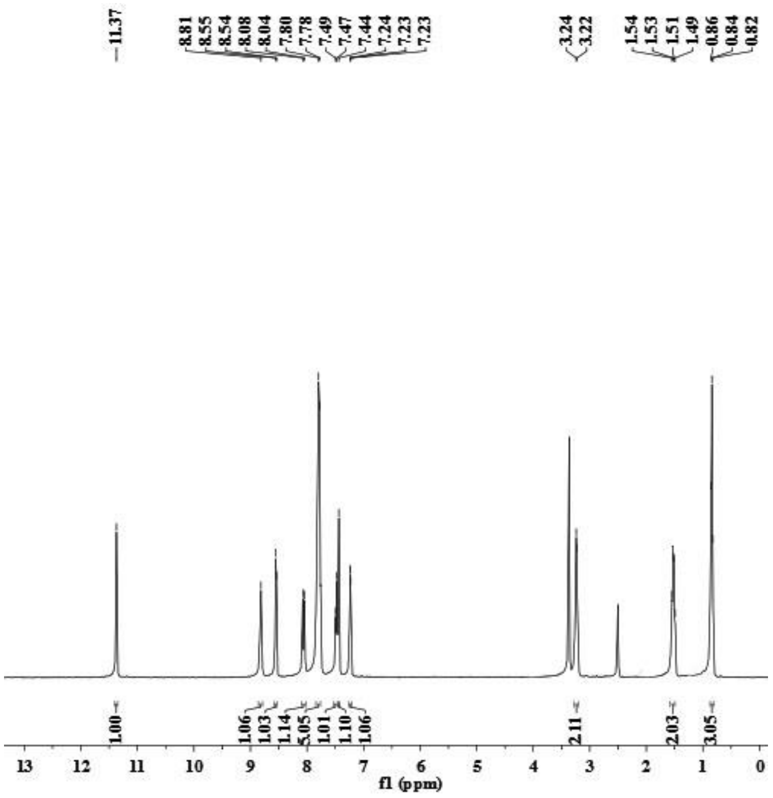

Figure 4. ¹H-NMR spectrum of compound **B13**

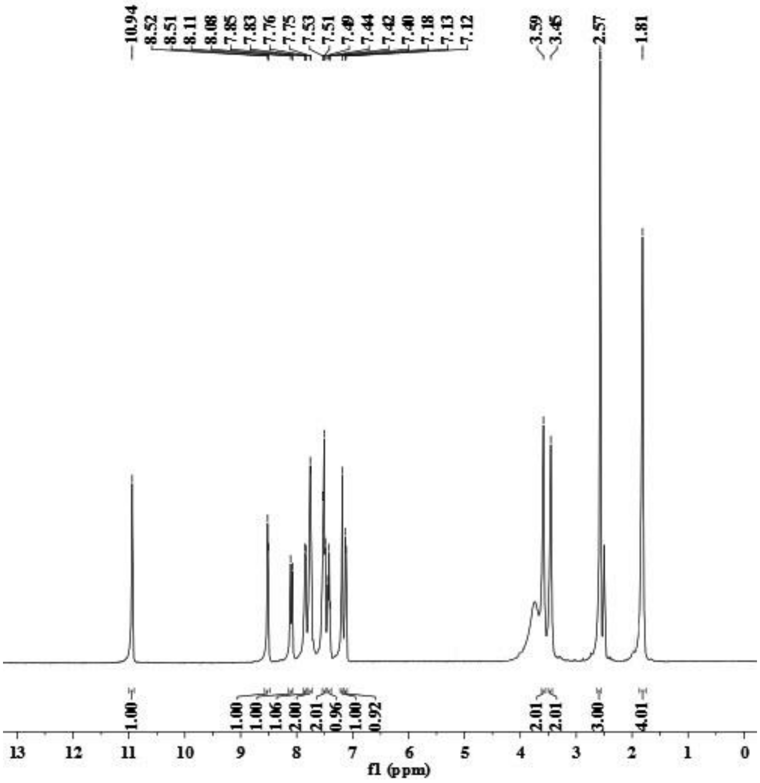

Figure 5. ¹H-NMR spectrum of compound **B20**

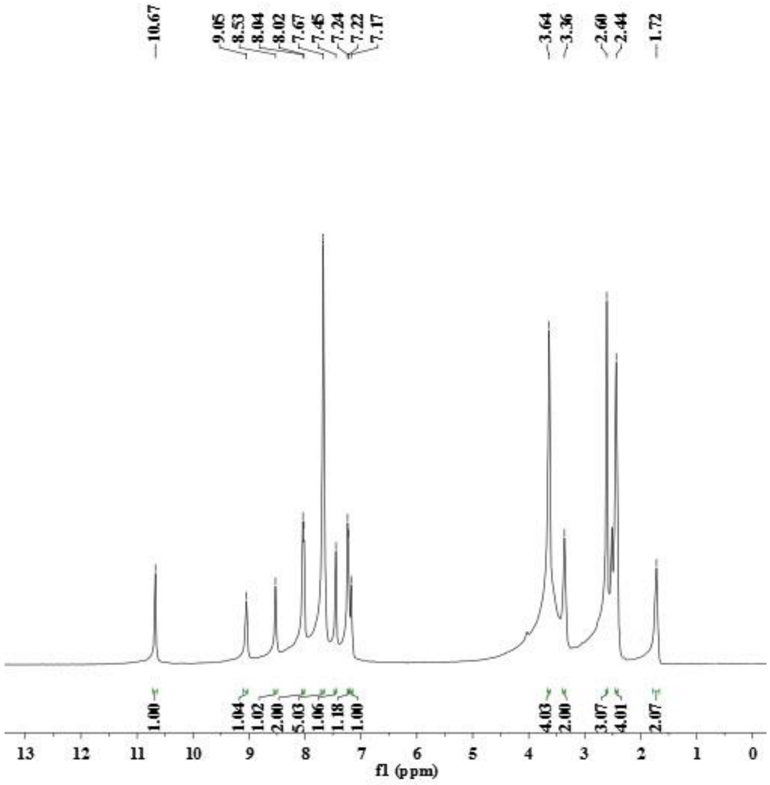

Figure 6. ^{1}H-NMR spectrum of compound **B25**

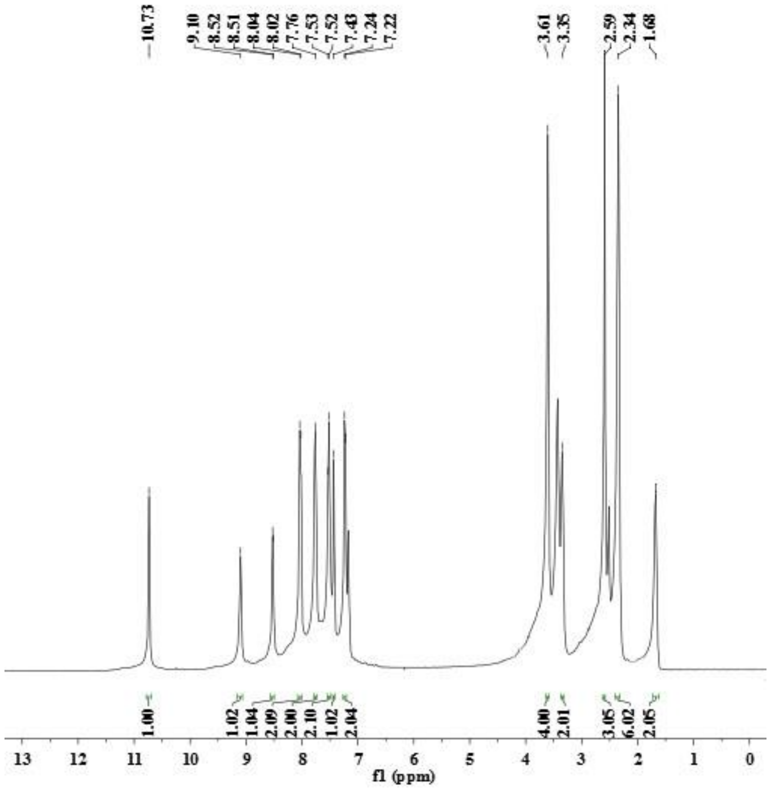

Figure 7. ^{1}H-NMR spectrum of compound **B26**

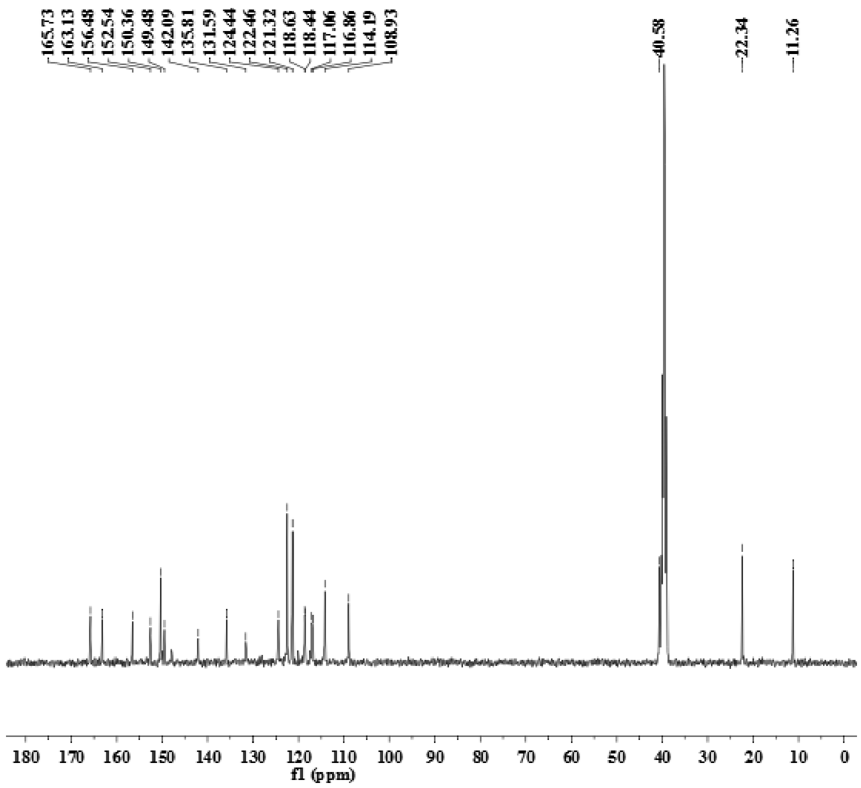

Figure 8. ^{13}C-NMR spectrum of compound **B6**

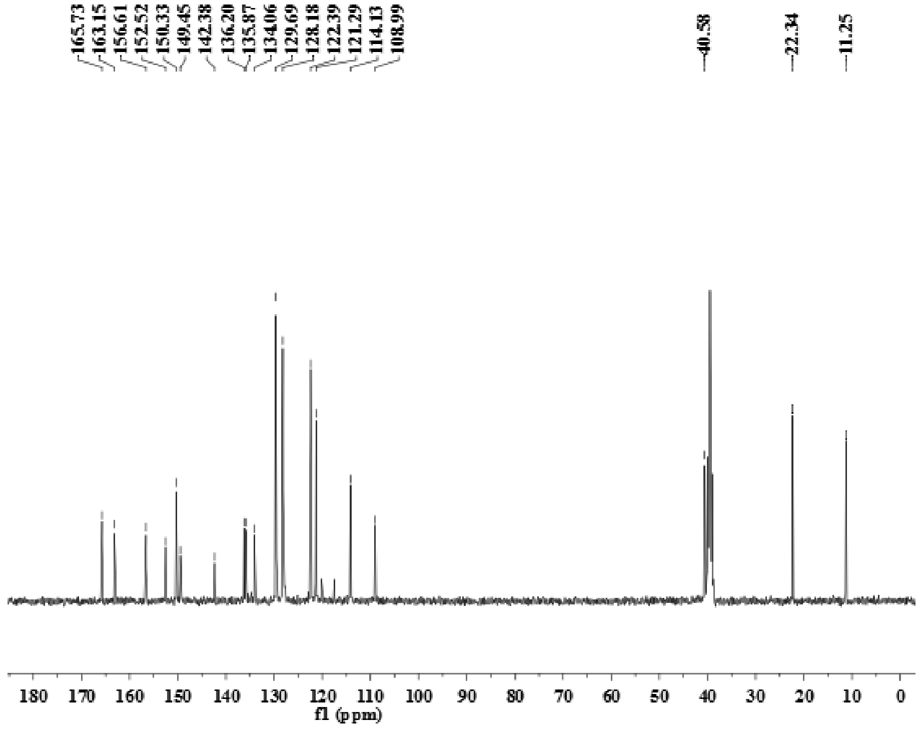

Figure 9. ^{13}C-NMR spectrum of compound **B7**

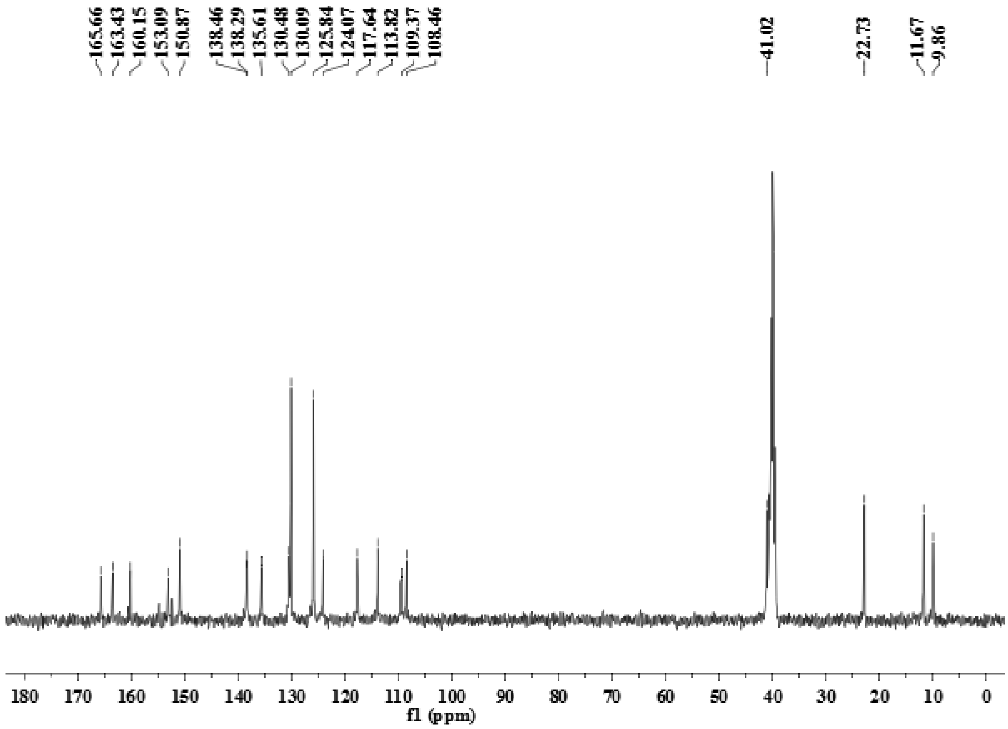

Figure 10. ^{13}C-NMR spectrum of compound **B10**

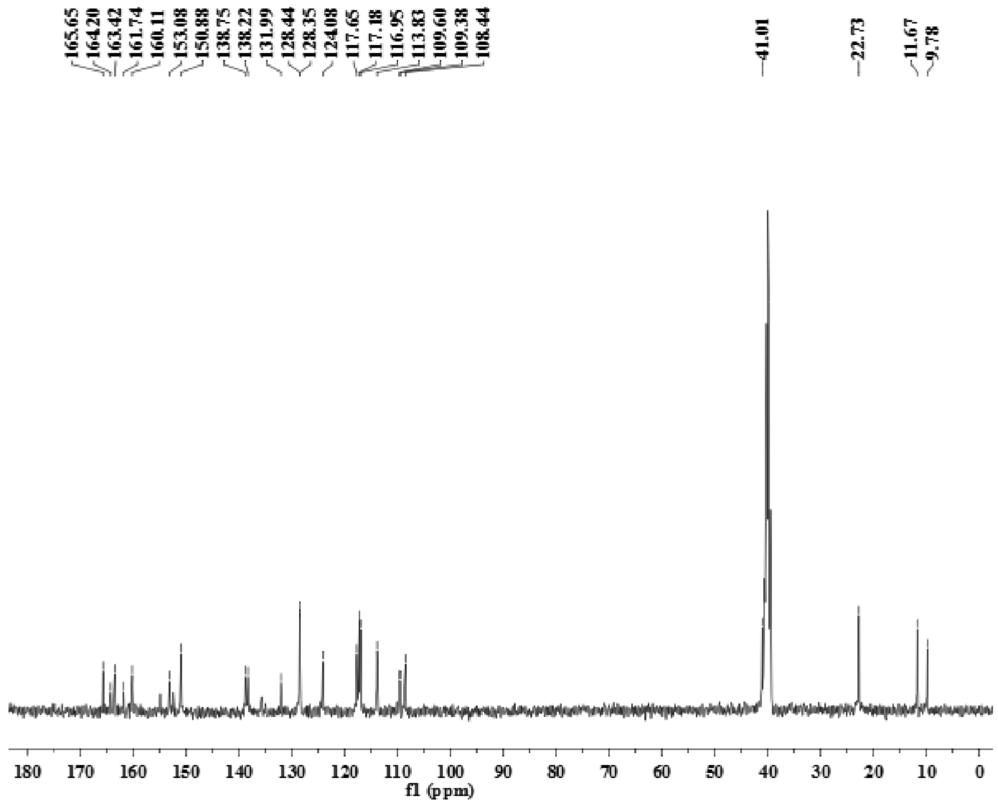

Figure 11. ^{13}C-NMR spectrum of compound **B11**

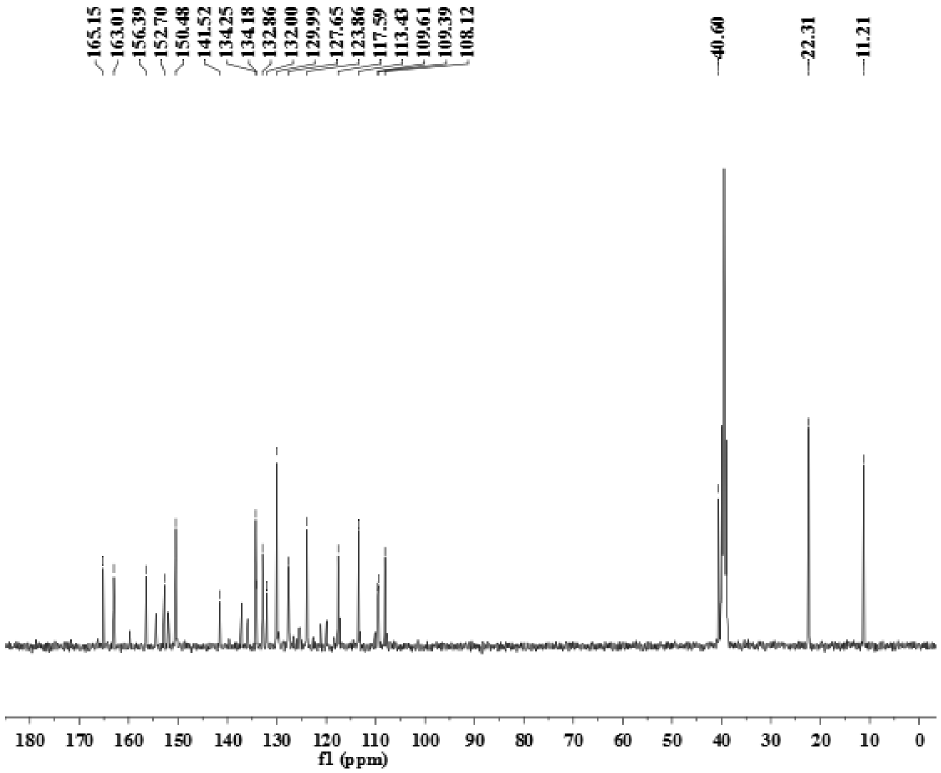

Figure 12. ^{13}C-NMR spectrum of compound **B14**

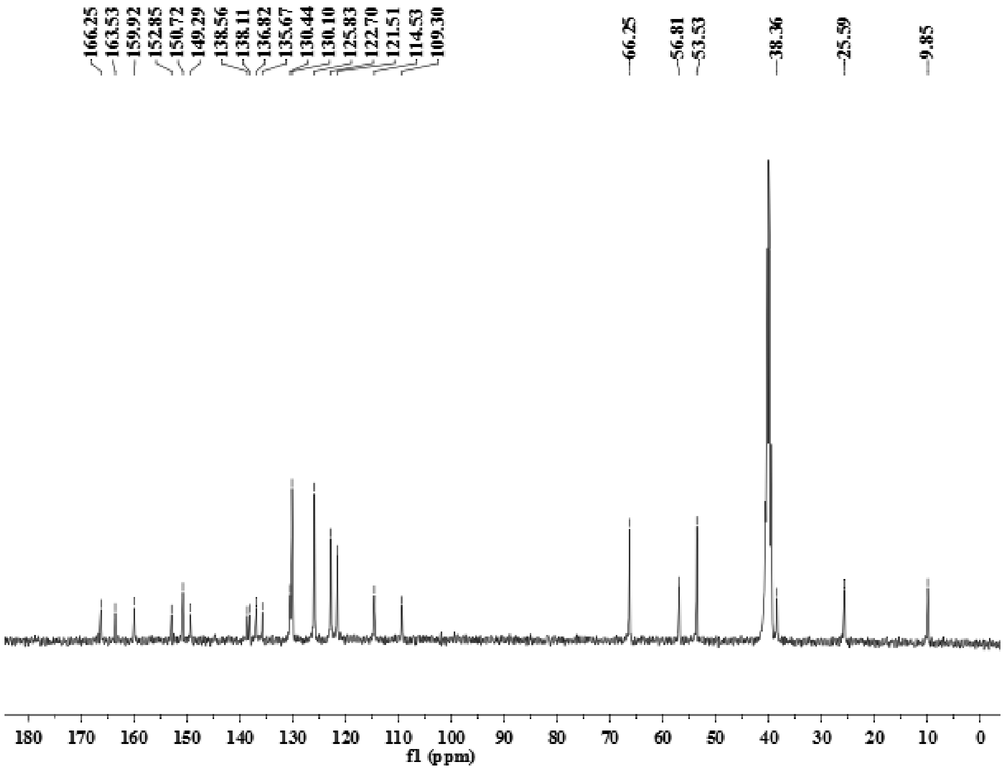

Figure 13. ^{13}C-NMR spectrum of compound **B25**

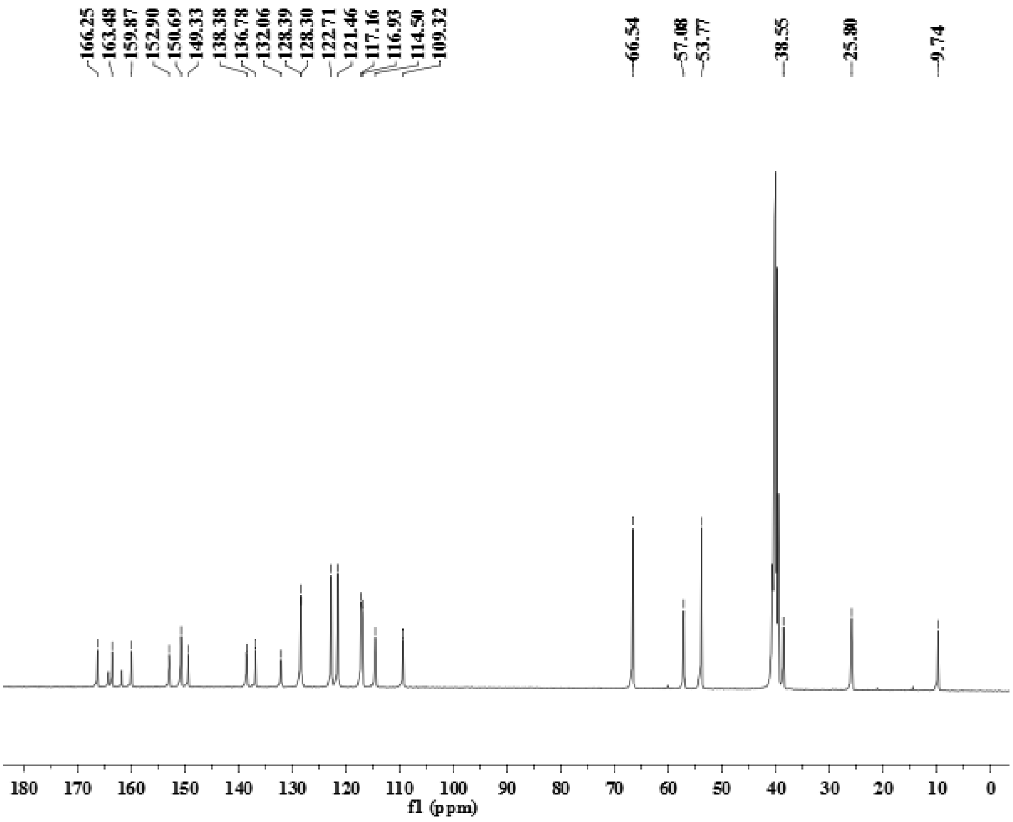

Figure 14. ^{13}C-NMR spectrum of compound **B26**

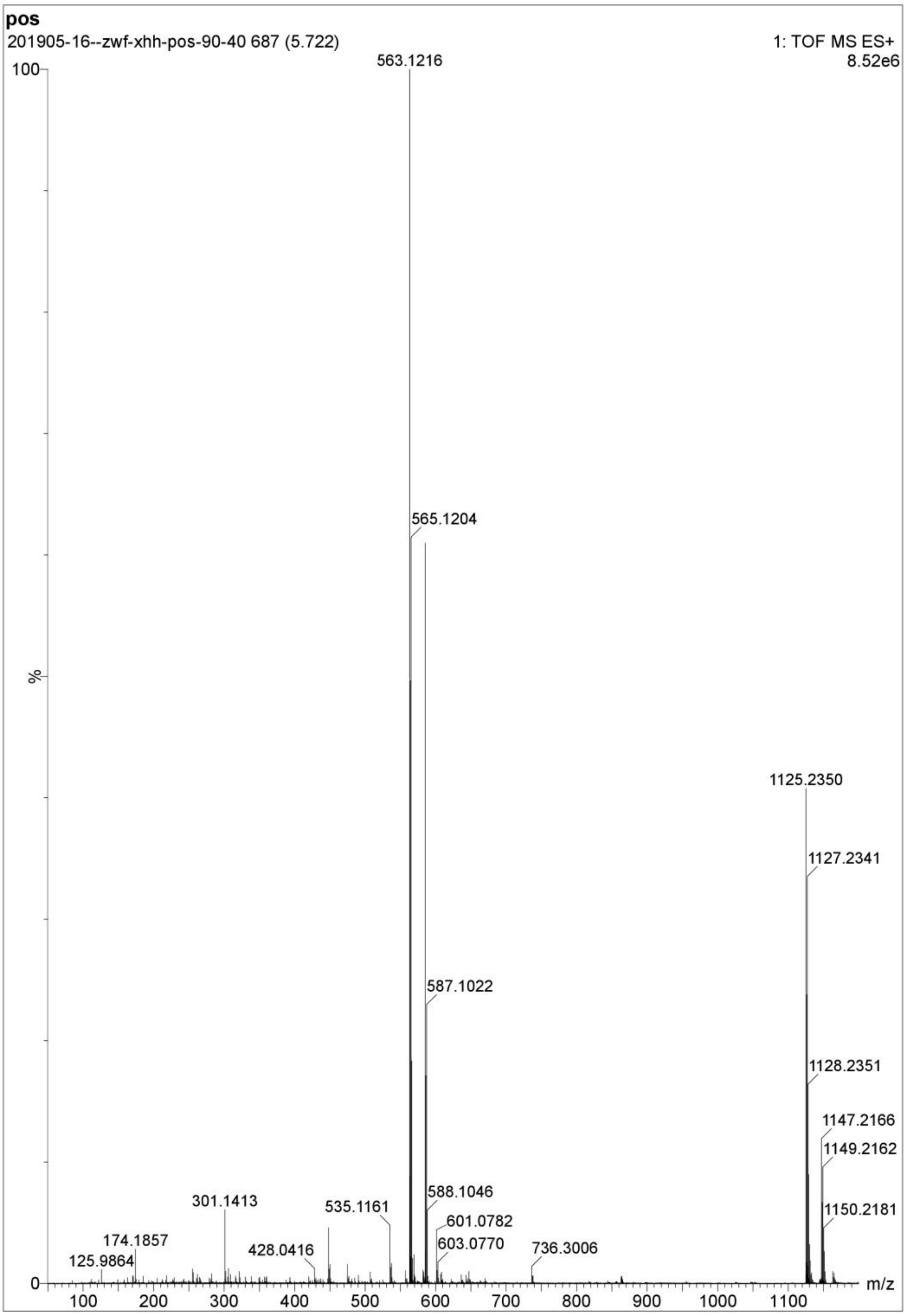

Figure 15. TOF MS of compound **B13**.

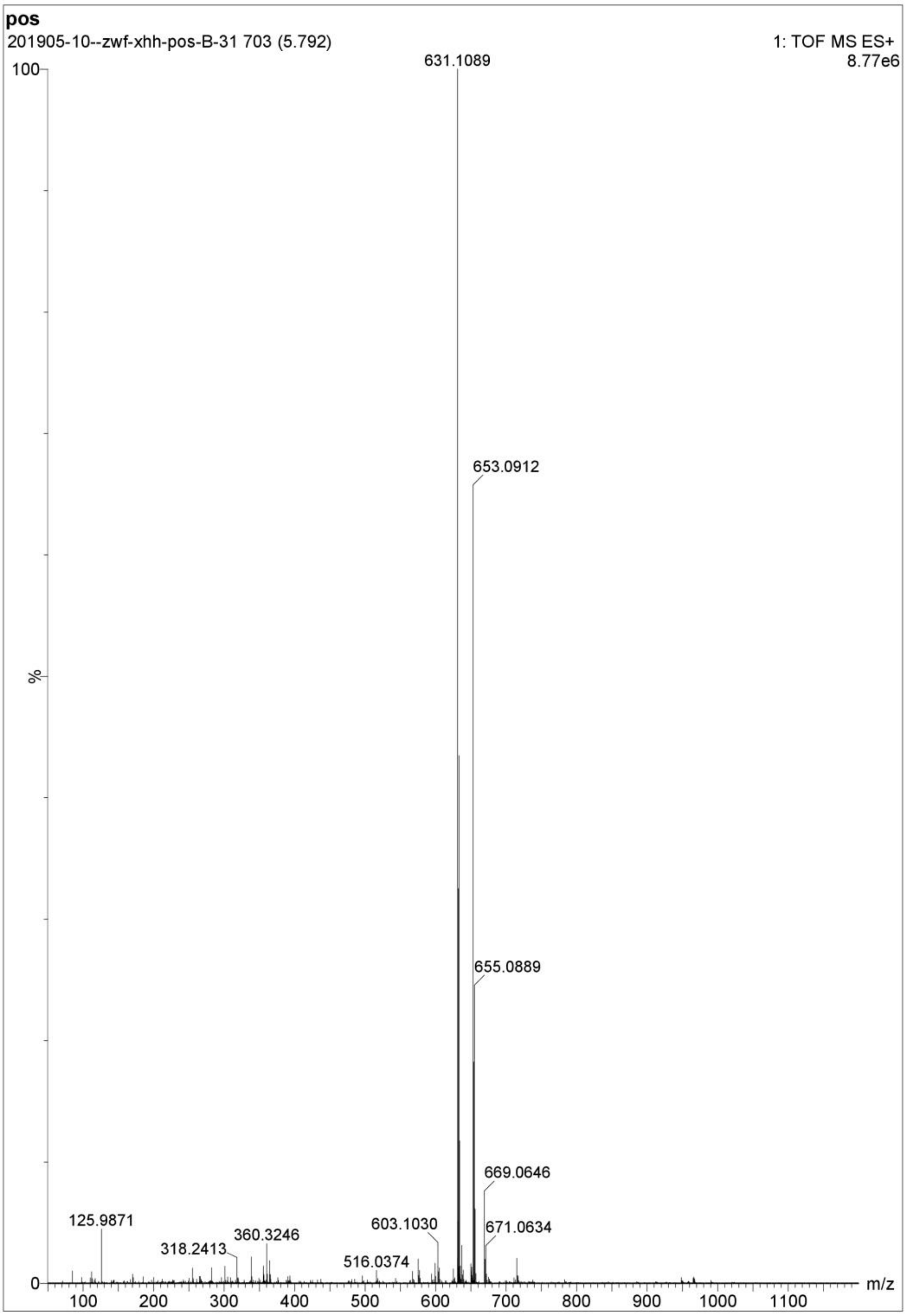

Figure 16. TOF MS of compound **B17**.

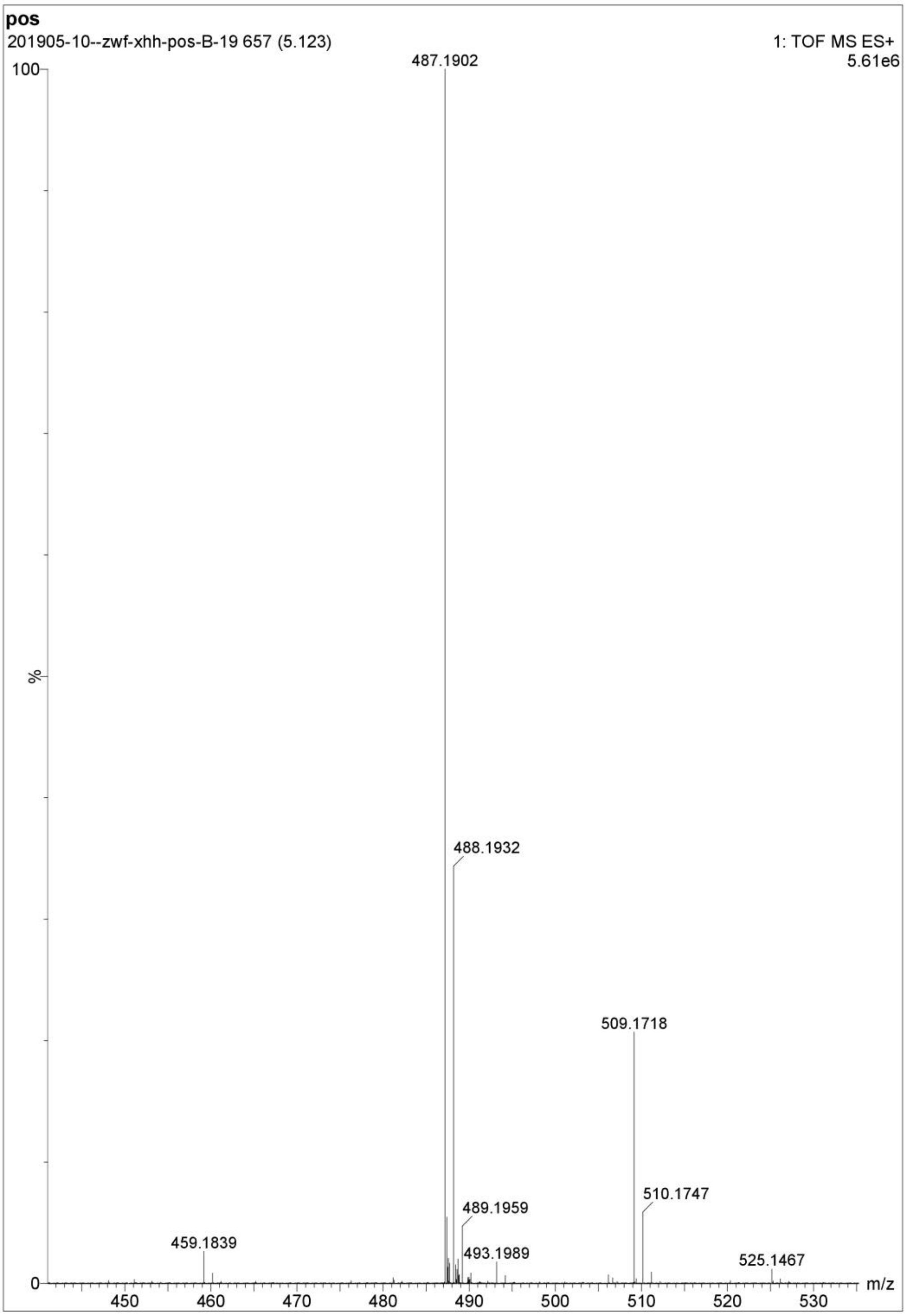

Figure 17. TOF MS of compound **B19**

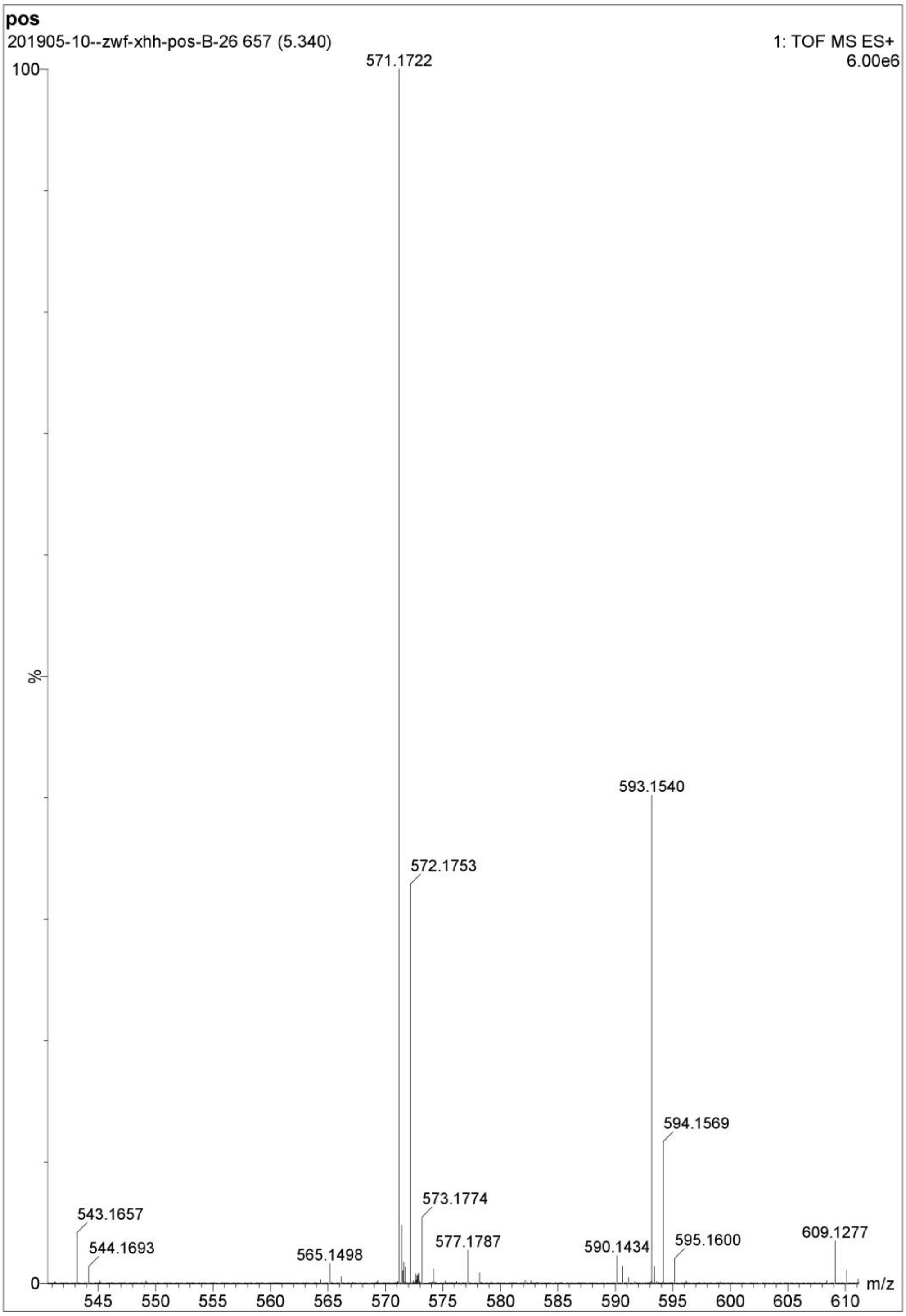

Figure 18. TOF MS of compound **B21**.

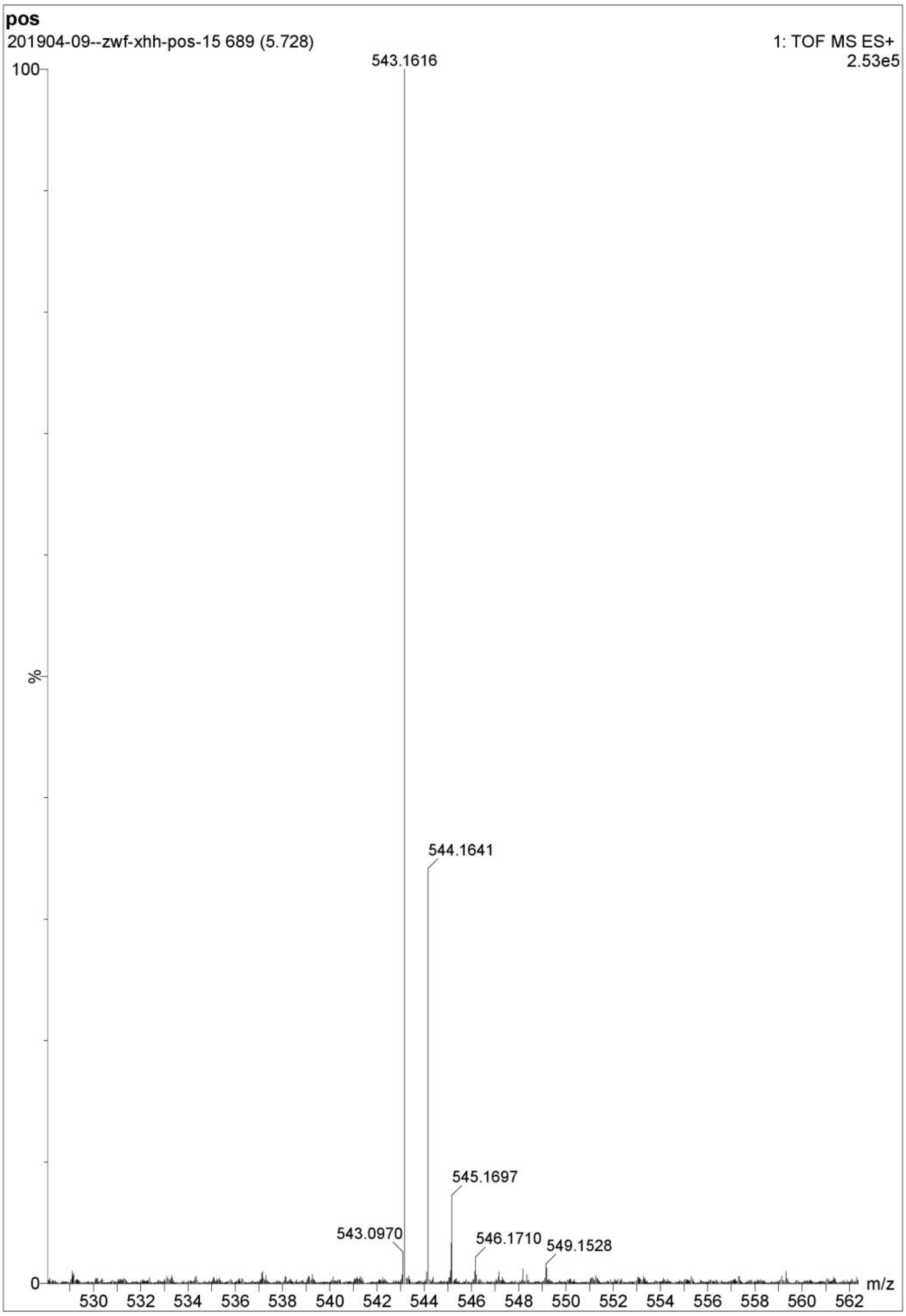

Figure 19. TOF MS of compound **B22**.

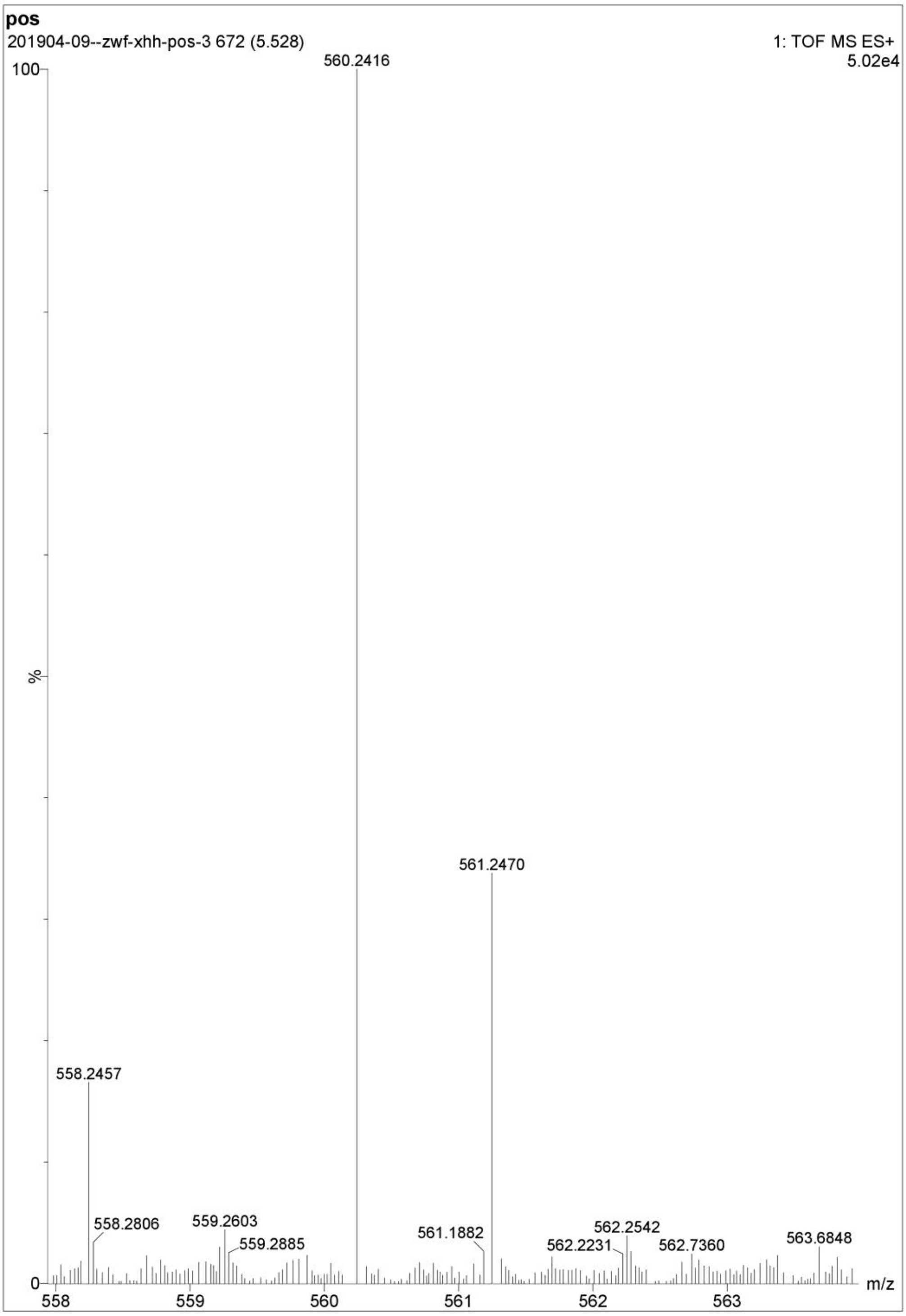

Figure 20. TOF MS of compound **B25**.
